# Nutzung von elektronenarmen Iminiumintermediaten zur Synthese von wertvollen Aminen

**DOI:** 10.1002/ange.202115435

**Published:** 2022-03-16

**Authors:** Che‐Sheng Hsu, Carlos R. Gonçalves, Veronica Tona, Amandine Pons, Marcel Kaiser, Nuno Maulide

**Affiliations:** ^1^ Institut für Organische Chemie Universität Wien Währinger Strasse 38 1090 Wien Österreich; ^2^ Schweizerisches Tropen- und Public-Health-Institut Socinstrasse 57 4002 Basel Schweiz

**Keywords:** Amine, Hydroaminoalkylierung, Iminiumionen, Synthetische Methoden, Säure-Aktivierte Reaktionen

## Abstract

Die Entwicklung von Reaktionen zur Umwandlung von Alkenen und Alkinen in wertvolle Bausteine ist nach wie vor eines der Hauptziele in der synthetischen Chemie. In dieser Arbeit berichten wir von der Nutzbarmachung von stark elektronenarmen, seltenen und kurzlebigen Iminiumionen zur Synthese von Aminen. Dies ermöglicht die milde Herstellung von Aminen mit z. B. trifluormethylierten Einheiten. Dieses umfassende Konzept wird durch die Aminierung von Chinin zu einem biologisch interessanten neuen Analogon verdeutlicht.

## Graphical abstract

Die Umwandlung von Alkenen und Alkinen in wertvolle Bausteine bleibt eines der Hauptziele der synthetischen Chemie. In unserer Arbeit stellen wir die Nutzung von stark elektronenarmen Iminiumionen für eine allgemeine und milde Aminsynthese vor, die die Herstellung von Produkten mit z. B. wertvollen α‐Trifluormethylgruppen ermöglicht. Dieses Konzept wird durch die Aminierung des Naturstoffs Chinin, und die damit einhergehende Synthese eines neuen biologisch interessanten Analogons, veranschaulicht.

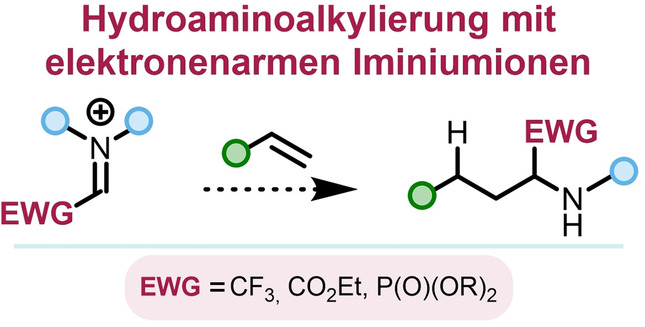

Iminiumionen sind ein priviligiertes Motiv in der Synthese von Aminen,^[1]^ und finden Anwendung in Prozessen wie der Mannich‐Reaktion,^[2]^ der reduktiven Aminierung^[3]^ oder auch nukleophilen Additionen.[^4^, ^5^, ^6^] Im Jahr 2018 berichteten Doyle et al. über eine Dreikomponenten‐Kupplung von Aminen, Arylaldehyden und Bromiden/Triflaten, die tertiäre Amine über eine nickelkatalysierte Reduktion eines Iminium‐Zwischenprodukts liefert, das in situ durch Kondensation erzeugt wird (Abbildung 1a).^[7]^ Noch im selben Jahr entwickelte die Gruppe von Gaunt ein photokatalytisches Protokoll für eine Mehrfachkomponenten‐Hydroaminoalkylierung (Abbildung 1b).^[8]^ Die Reaktion wurde auf die Totalsynthese von (−)‐FR901483 und (+)‐TAN1251C angewandt und verläuft durch SET‐Reduktion (SET=single‐electron transfer) eines transienten Iminiumions, das wiederum in situ durch Kondensation eines Amins mit einem Aldehyd oder Keton erzeugt wird.^[9]^ Letztes Jahr berichtete die gleiche Gruppe von Bedingungen für eine radikalbasierte Synthese von Aminen über die radikalische Addition an Iminiumintermediate (Abbildung 1c).^[10]^ Es ist hervorzuheben, dass alle aktuellen Methoden den Einsatz von stöchiometrischen Mengen an Reduktionsmitteln erfordern (Abbildung 1).

**Figure 1:**
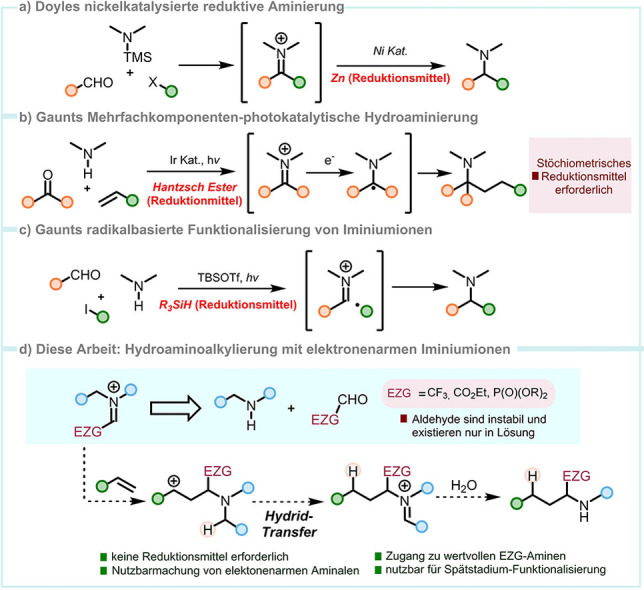
a,b,c) Moderne Methoden für die Synthese von Aminen. d) Entwicklung einer metallfreien Hydroaminoalkylierung für Olefine und Alkine.

Die Verwendung des Ansatzes der Kondensation mit Carbonylen zur Erzeugung des entscheidenden Iminiumintermediats gibt zwar ein gewisses Maß an Flexibilität, beeinträchtigt aber dessen Einsatz für elektronenarme Iminiumionen wie jenen in Abbildung 1d. Tatsächlich sind solche Spezies kaum in der Literatur beschrieben,[^11^, ^12^, ^13^] insbesondere in der Verbindung mit inaktivierten Alkenen. Das ist wenig überraschend, da viele Aldehyde, wie zum Beispiel 2,2,2‐Trifluoracetalaldehyd, flüchtig sind und oft nur als die entsprechenden Hydrate/Halbacetale erhältlich sind. Dennoch ist die Möglichkeit des Zugangs zu Aminierungsprodukten, die eine CF_3_‐Gruppe tragen, aufgrund deren interessanten Eigenschaften sowohl für die Arzneimittelforschung als auch für die Materialwissenschaften von potenziell enormem Wert.[^14^, ^15^, ^16^]

Wir zogen einen anderen Ansatz für die Erzeugung der reaktiven Intermediate in Betracht, nämlich die in situ Herstellung aus stabilen Aminalvorstufen.[^17^, ^18^, ^19^] Es ist bemerkenswert, dass solche Zwischenstufen den Zugang zu einer Reihe von hochfunktionalisierten Aminen ermöglichte, welche mit keiner davor genannten Methoden zugänglich waren (Abbildung 1d).

In dieser Arbeit präsentieren wir die Nutzung von elektronenarmen Iminiumionen in der Synthese von trifluormethylierten Aminen, Aminoestern und Aminophosphonaten durch Hydroaminoalkylierung von nicht aktivierten Alkenen und Alkinen. Unser Konzept basiert auf der Paarung einer schnellen intermolekularen aza‐Prins‐artigen Reaktion mit elektronenarmen Spezies mit einem stereoselektiven internen Reduktionsereignis (1,5‐Hydridtransfer),[^18^, ^20^, ^21^, ^22^, ^23^] das sicherstellt, dass die C−C Bindungsbildung in einer redox‐neutralen Weise und nicht, wie vorher beschrieben Methoden, unter Verbrauch externer Reduktionsmittel stattfindet (Abbildung 1d). Diese Strategie ermöglicht die breite Herstellung wertvoller Amine und die späte Funktionalisierung komplexer Strukturen.

Wir fokussierten unsere anfänglichen Untersuchungen auf das kommerziell erhältliche, CF_3_‐substituierte Aminal **A** (siehe Abbildung 2), ursprünglich entwickelt von Dolbier für die Synthese von propargylischen und allylischen α‐Trifluormethylaminen,^[11]^ und betrachteten es als ideale Plattform um unsere Hypothese zu untersuchen. Schnell fanden wir heraus, dass die erhöhte Elektrophilie und Reaktivität von **A** ausgesprochen milde Bedingungen erlaubte. Insbesondere bei tiefen Temperaturen (−10 °C) beobachteten wir, dass die Kombination von **A** mit nicht‐aktivierten Alkenen die Bildung der gewünschten sekundären α‐Trifluormethylamine ermöglichte (Abbildung 2A; siehe Hintergrundinformationen für die vollständige Optimierung). Da bekannte Methoden zur Herstellung von α‐Trifluormethylaminen oft mehrstufige Verfahren darstellen[^24^, ^25^, ^26^, ^27^, ^28^, ^29^, ^30^, ^31^, ^32^, ^33^] oder auf instabilen Reagenzien unter oxidativen Bedingungen[^34^, ^35^, ^36^] beruhen, bietet die Kupplung von **A** mit leicht verfügbaren Olefin‐Ausgangsmaterialien eine direkte Lösung für diese Syntheseherausforderung. Das Anwendungsgebiet dieser Reaktion erwies sich als besonders groß. Sowohl lineare (**1 a**, **1 aa**) als auch cyclische Alkene (**3 a**, **4 a**) wurden in guten bis ausgezeichneten Ausbeuten in die α‐trifluormethylaminierten Produkte umgewandelt (Abbildung 2A). Dabei wurde eine hohe Toleranz gegenüber funktionellen Gruppen beobachtet, darunter Halogenide (**5 a**), Ester (**6 a**) und sogar freie Alkohole (**7 a**). Alkine reagierten ebenfalls problemlos und stereoselektiv, und lieferten isomerenreine di‐ und trisubstituierte olefinische Produkte (**8 a**, **9 a** und **11 a**).[^12^, ^37^] Ein Enin‐Substrat zeigte große Chemoselektivität für die Dreifachbindung (**10 a**). Zusätzlich wurde festgestellt, dass ein terminales Alkin bevorzugt zu einem internen reagiert (**12 a**).

**Figure 2:**
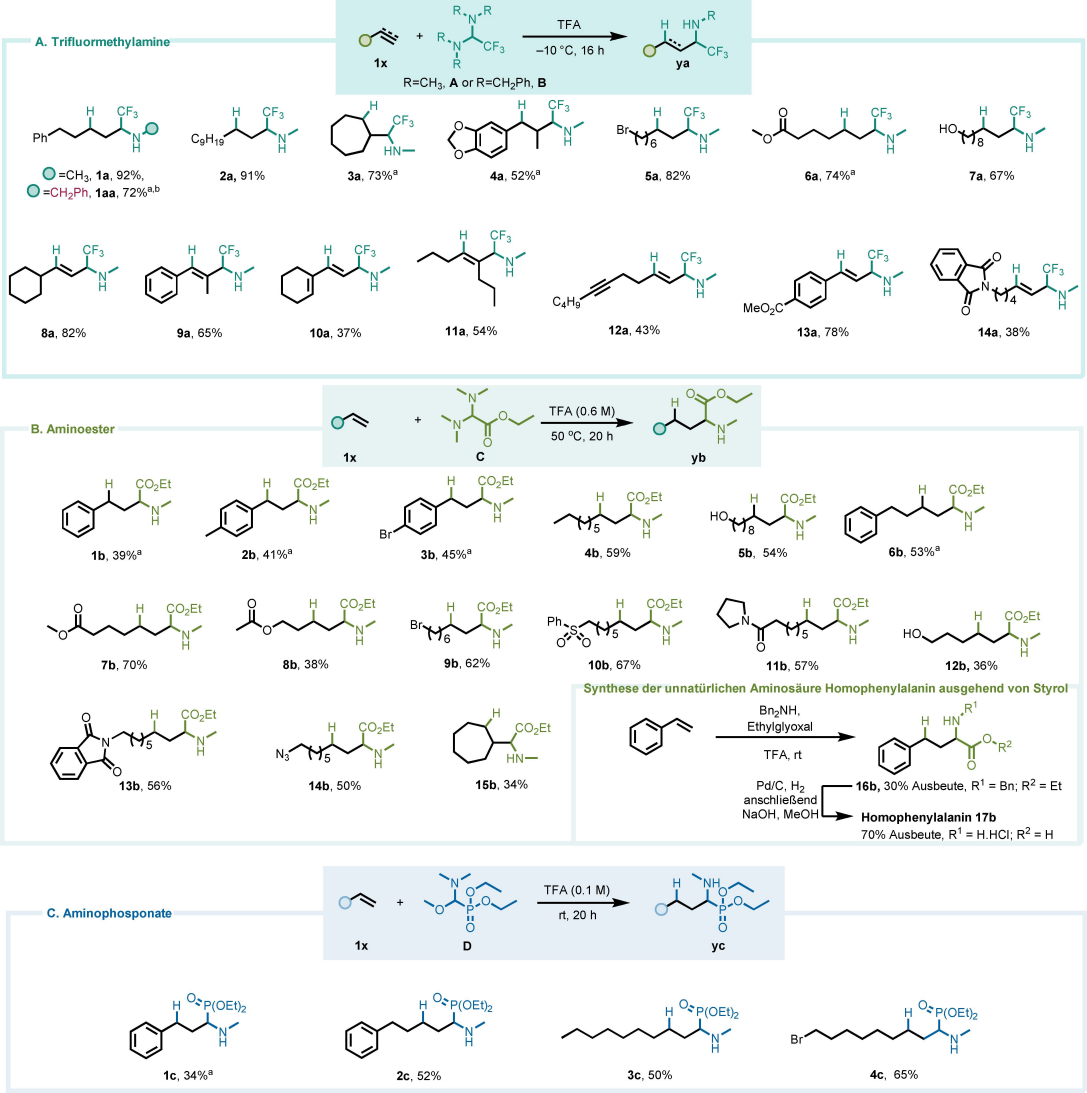
Substratbreite für die Synthese von α‐Trifluormethyl‐, α‐Carboethoxy‐ und α‐Phosphonylaminen durch Hydroaminoalkylierung von Alkenen und Alkinen. [a] Reaktion bei 20 °C durchgeführt. [b] Reaktion unter Verwendung von in situ erzeugtem Aminal **B** anstelle von **A** – Siehe Hintergrundinformationen für Details.

Ein bemerkenswertes Beispiel ist in **1 aa** dargestellt, in dem ein in situ erzeugtes Benzylaminal **B** verwendet wurde, um ein Benzylaminprodukt zu erhalten. Dieses Ergebnis unterstreicht noch einmal die Praktikabilität der Methode, da vielfältig substituierte Aminale schnell synthetisiert und ohne weitere Reinigung verwendet werden können.

Nachdem wir den Umfang möglicher Substrate für die Trifluormethylaminoalkylierung untersucht hatten, richteten wir unsere Aufmerksamkeit auf verschiedene stark elektronenarme Iminiumionen. Insbesondere erkannten wir den Reiz einer beispiellosen, direkten Bildung von α‐Aminosäurederivaten aus nicht‐aktivierten Alkenen. Dies ist eine seit langem angestrebte Umwandlung in der organischen Synthese, wobei eine direkte, einstufige Strategie bis dato noch ausstand.^[38]^

Tatsächlich erwies sich Carbethoxyaminal^[39]^ **C** (Abbildung 2B) unter leichtem Erhitzen als geeignet für diese Aufgabe (siehe Hintergrundinformationen für die vollständige Optimierung). Elektronenreiche (**1 b**, **2 b**) und ‐arme Styrole (**3 b**) zeigten eine vergleichbar gute Leistung und wurden in mäßigen Ausbeuten in die entsprechenden α‐Aminosäurederivate umgewandelt. Die Anwesenheit funktioneller Gruppen beeinträchtigte auch diese Reaktion nicht, und so war es möglich, α‐Aminoester mit einer Hydroxyl‐ (**5 b**), einer Ester‐ (**7 b**) oder einer Sulfoneinheit (**10 b**) entlang der aliphatischen Kette zu bewahren.

Selbst ein primäres Bromid (**9 b**) ging unter den angegebenen Bedingungen keine nukleophile Substitution ein, und auch ein Azid (**14 b**) blieb unberührt. Als Anwendungsbeispiel ermöglicht die vorgestellte Methode eine unkomplizierte Synthese der nicht‐natürlichen α‐Aminosäure Homophenylalanin (**17 b**) aus Styrol in nur drei Stufen. Wie gezeigt, kann die Hydroaminoalkylierung in diesem Fall als 3‐Komponenten‐Kupplung mit einem sekundären Amin, einem Glyoxalat und einem Olefin durchgeführt werden (Abbildung 2B).

Schließlich stellten wir mit Blick auf α‐Aminophosphonate als Ziel fest, dass die Acidolyse des Halbaminals **D** (Abbildung 2C) ebenfalls zur selektiven Bildung einer Iminiumspezies führte, die für die redoxneutrale Kupplung mit Alkenen geeignet ist.^[40]^ Wie in den vorangegangenen Fällen lieferten sowohl Styrole als auch aliphatische Alkene die gewünschten Addukte (**1 c**–**4 c**) in guten Ausbeuten.

Die hier dargelegten Verfahren bieten Möglichkeiten zur Anwendung in biologischen Fragestellungen. Wie in Abbildung 3A und B gezeigt, konnte ein trifluormethyliertes Derivat des Antihistaminikums Chlorpheniramin der 1. Generation schnell in einer Tandem‐Hydroaminoalkylierung/Eschweiler‐Clarke‐Reaktion (**16 a**) hergestellt werden. Auf ähnliche Weise wurde auch ein Analogon des berühmten Antimalaria‐Medikaments Chloroquin (**15 a**) erfolgreich synthetisiert. Der Naturstoff Chinin wurde, in einem interessanten Beispiel einer späten Funktionalisierung, ebenfalls erfolgreich in ein α‐Trifluormethylamin‐Derivat (**17 a**) umgewandelt. Aufgrund der bekannten Antimalariaaktivität von Chinin[^41^, ^42^] waren wir von diesem Analogon und seinem Potenzial als Antimalariamittel besonders angetan. Das Chinin‐Analogon **17 a** wurde verwendet, um zwei unterschiedliche Zelllinien zu behandeln, wobei die erste P. falciparum und die zweite L6‐Rattenzellen enthielt, um so die Antimalaria‐Aktivität bzw. Zytotoxizität zu überprüfen. **17 a** erzielte eine starke in vitro‐Hemmung (IC_50_=24 nM) des Wachstums von P. falciparum und die Bewertung seiner Wirkung auf L6‐Rattenzellen zeigte, dass es nicht zytotoxisch ist. Wichtig ist, dass die Antimalariaaktivität von **17 a** mit der von Chloroquinsulfat vergleichbar ist, einem Medikament, das zur Behandlung ähnlicher Krankheiten eingesetzt wird.^[43]^

**Figure 3:**
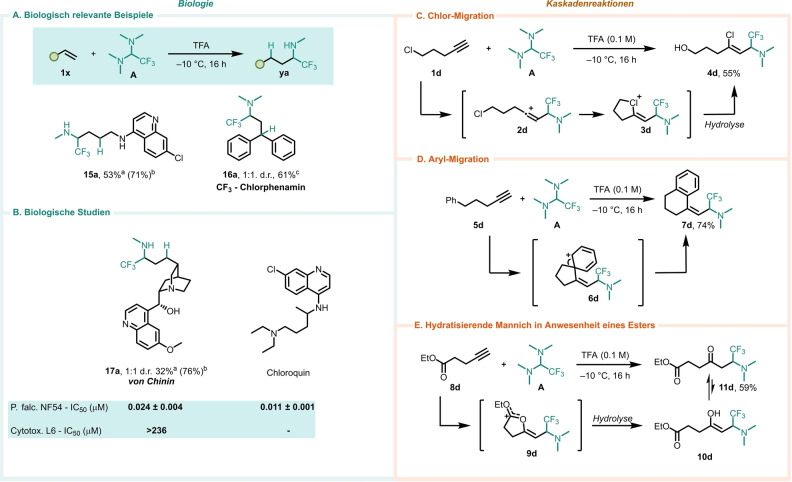
A, B) Ausgewählte Anwendungen auf biologische Probleme. C–E) Mechanistische Experimente. [a] Reaktion durchgeführt bei 75 °C. [b] Basierend auf wiedergewonnenem Ausgangsmaterial. [c] Mit 10 equiv von Paraformaldehyd und HCOOH bei 100 °C – Siehe Hintergrundinformationen für Details.

Letztlich bietet die Integration dieser Reaktionen in interessante Dominoprozesse nützliche Synthesemöglichkeiten (Abbildung 3C–E). Beispielsweise ermöglichen Halogen‐ oder Arenmigrationsprozesse die Umwandlung relativ einfacher (Alkin **5 d**) und kommerziell erhältlicher (5‐Chlorpentin (**1 d**)) Ausgangsmaterialien, in aminierte Produkte höherer Komplexität (Abbildung 3C und D). Die strategische Positionierung einer Estereinheit (**8 d**) führt zum Abfang des intermediär gebildeten Kations und liefert Produkte einer formalen hydratisierenden Mannich‐Transformation (Abbildung 3E).

Wir haben hierin einen Ansatz vorgestellt, der stark elektronenarme Iminiumionen nutzt, um die Kupplung mit nicht‐aktivierten ungesättigten Partnern zu ermöglichen, ohne dass ein Reduktionsmittel erforderlich ist. Dabei bieten wir einstufige Synthesewege zu trifluormethylierten Aminen, Aminoestern und Aminophosphonaten von potenziell hohem Wert für die medizinische und pharmazeutische Forschung, sowie die Materialchemie. Die beschriebene späte Derivatisierung von Chinin legt eine allgemeine strategische Anwendung dieser Reaktion auf bioaktive Substanzen nahe, die eine Doppelbindung tragen.

## Interessenkonflikt

Die Autoren erklären, dass keine Interessenkonflikte vorliegen.

## Supporting information

As a service to our authors and readers, this journal provides supporting information supplied by the authors. Such materials are peer reviewed and may be re‐organized for online delivery, but are not copy‐edited or typeset. Technical support issues arising from supporting information (other than missing files) should be addressed to the authors.

Supporting Information

## Data Availability

Die Daten, die die Ergebnisse dieser Studie unterstützen, sind auf begründete Anfrage beim Autor erhältlich.

## References

[1] A. Trowbridge , S. M. Walton , M. J. Gaunt , Chem. Rev. 2020, 120, 2613–2692. 32064858 10.1021/acs.chemrev.9b00462

[2a] S. Saranya , N. A. Harry , K. K. Krishnan , G. Anilkumar , Asian J. Org. Chem. 2018, 7, 613–633;

[2b] B. Biersack , K. Ahmed , S. Padhye , R. Schobert , Expert Opin. Drug Discovery 2018, 13, 39–49. 10.1080/17460441.2018.1403420 29137490

[3a] O. I. Afanasyev , E. Kuchuk , D. L. Usanov , D. Chusov , Chem. Rev. 2019, 119, 11857–11911; 31633341 10.1021/acs.chemrev.9b00383

[3b] C. Wang , J. Xiao , Top. Curr. Chem. 2013, 343, 261–282. 10.1007/128_2013_484 24158548

[4] Für aktuelle Beispiele zu nukleophilen Additionen an Iminiumionen, siehe:

[4a] D. B. Freeman , L. Furst , A. G. Condie , C. R. J. Stephenson , Org. Lett. 2012, 14, 94–97; 22148974 10.1021/ol202883v PMC3253246

[4b] A. V. Tsymbal , M. D. Kosobokov , V. V. Levin , M. I. Struchkova , A. D. Dilman , J. Org. Chem. 2014, 79, 7831–7835; 25116859 10.1021/jo501644m

[4c] A. E. Hartman , C. L. Brophy , J. A. Cupp , D. K. Hodge , T. J. Peelen , J. Org. Chem. 2009, 74, 3952–3954; 19366203 10.1021/jo8027714

[4d] E. L. Myers , J. G. de Vries , V. K. Aggarwal , Angew. Chem. Int. Ed. 2007, 46, 1893–1896; 10.1002/anie.200604715 17278162

[5a] G. Blay , A. Monleón , J. R. Pedro , Curr. Org. Chem. 2009, 13, 1498–1539;

[5b] Q.-H. Zheng , W. Meng , G.-J. Jiang , Z.-X. Yu , Org. Lett. 2013, 15, 5928–5931. 24237286 10.1021/ol402517e

[6a] T. Thaima , F. Zamani , C. J. T. Hyland , S. G. Pyne , Synthesis 2017, 49, 1461–1480;

[6b] P. Wu , M. Givskov , T. E. Nielsen , Chem. Rev. 2019, 119, 11245–11290. 31454230 10.1021/acs.chemrev.9b00214 PMC6813545

[7] C. Heinz , J. P. Lutz , E. M. Simmons , M. M. Miller , W. R. Ewing , A. G. Doyle , J. Am. Chem. Soc. 2018, 140, 2292–2300. 29341599 10.1021/jacs.7b12212 PMC6698183

[8] A. Trowbridge , D. Reich , M. J. Gaunt , Nature 2018, 561, 522–527. Für aktuelle Beispiele zu metallkatalysierter Hydroaminoalkylierung, siehe:30258135 10.1038/s41586-018-0537-9

[8a] J. Yang , F. G. Delolo , A. Spannenberg , R. Jackstell , M. Beller , Angew. Chem. Int. Ed. 2022, 61, e202112597; 10.1002/anie.202112597 PMC9299624 34738697

[8b] T. Kaper , M. Fischer , M. Warsitz , R. Zimmering , R. Beckhaus , S. Doye , Chem. Eur. J. 2020, 26, 14300–14304. 32844473 10.1002/chem.202003484 PMC7702142

[9] D. Reich , A. Trowbridge , M. J. Gaunt , Angew. Chem. Int. Ed. 2020, 59, 2256–2261; 10.1002/anie.201912010 31693285

[10] R. Kumar , N. J. Flodén , W. J. Whitehurst , M. J. Gaunt , Nature 2020, 581, 415–421. 32268340 10.1038/s41586-020-2213-0 PMC7116815

[11] Y. Xu , X. X. Rong , W. R. Dolbier , J. Org. Chem. 1997, 62, 1576–1577. 10.1021/jo970927q 11671875

[12] Y. Xu , W. R. Dolbier , J. Org. Chem. 2000, 65, 2134–2137. 10774037 10.1021/jo991750y

[13] Y. Xu , W. R. Dolbier , Tetrahedron Lett. 1998, 39, 9151–9154.

[14] J. Jin , C. M. Topping , S. Chen , J. Ballato , S. H. Foulger Jr , D. W. Smith , J. Polym. Sci. Part A 2004, 42, 5292–5300.

[15] K. Müller , C. Faeh , F. Diederich , Science 2007, 317, 1881–1886. 17901324 10.1126/science.1131943

[16] N. A. Meanwell , J. Med. Chem. 2018, 61, 5822–5880. 29400967 10.1021/acs.jmedchem.7b01788

[17] Für das seltene Beispiel der Anwendung von O,N- (eher als N,N)-Aminalen, siehe: Y.-Y. Huang , C. Cai , X. Yang , Z.-C. Lv , U. Schneider , ACS Catal. 2016, 6, 5747–5763.

[18] Für unsere frühere Arbeit mit unsubstituierten Aminalen, siehe: D. Kaiser , V. Tona , C. R. Gonçalves , S. Shaaban , A. Oppedisano , N. Maulide , Angew. Chem. Int. Ed. 2019, 58, 14639–14643; 10.1002/anie.201906910 PMC6790944 31482639

[19] Für frühere Arbeiten zu Iminium/Alken-Kupplungen siehe:

[19a] T. Cohen , A. Onopchenko , J. Org. Chem. 1983, 48, 4531–4537;

[19b] A. R. Ofial , H. Mayr , J. Org. Chem. 1996, 61, 5823–5830.

[20] M. C. Haibach , D. Seidel , Angew. Chem. Int. Ed. 2014, 53, 5010–5036; 10.1002/anie.201306489 24706531

[21] B. Peng , N. Maulide , Chem. Eur. J. 2013, 19, 13274–13287. 24027042 10.1002/chem.201301522

[22] B. Wang , D. A. Gandamana , D. F. L. Rayo , F. Gagosz , S. Chiba , Org. Lett. 2019, 21, 9179–9182. 31674788 10.1021/acs.orglett.9b03548

[23] X.-D. An , J. Xiao , Org. Chem. Front. 2021, 8, 1364–1383.

[24] S. G. K. Prakash , M. Mandal , G. A. Olah , Synlett 2001, 77–78.

[25] S. G. K. Prakash , Y. Wang , R. Mogi , J. Hu , T. Mathew , G. A. Olah , Org. Lett. 2010, 12, 2932–2935. 20518520 10.1021/ol100918d

[26] V. I. Supranovich , V. V. Levin , M. I. Struchkova , A. D. Dilman , Org. Lett. 2018, 20, 840–843. 29355326 10.1021/acs.orglett.7b03987

[27] A. A. S. Gietter-Burch , V. Devannah , D. A. Watson , Org. Lett. 2017, 19, 2957–2960. 28535057 10.1021/acs.orglett.7b01196 PMC5531277

[28] C. Alonso , M. González , M. Fuertes , G. Rubiales , J. M. Ezpeleta , F. Palacios , J. Org. Chem. 2013, 78, 3858–3866. 23485177 10.1021/jo400281e

[29] F. Gagosz , S. Z. Zard , Org. Lett. 2003, 5, 2655–2657. 12868882 10.1021/ol034812m

[30] Z. Neouchy , D. G. Pardo , J. Cossy , Org. Lett. 2018, 20, 6017–6021. 30216084 10.1021/acs.orglett.8b02353

[31] L.-M. Shi , X.-S. Sun , C. Shen , Z.-F. Wang , H.-Y. Tao , C.-J. Wang , Org. Lett. 2019, 21, 4842–4848. 31145631 10.1021/acs.orglett.9b01738

[32] C. I. Onyeagusi , S. J. Malcolmson , ACS Catal. 2020, 10, 12507–12536. 34306806 10.1021/acscatal.0c03569 PMC8302206

[33] C. I. Onyeagusi , X. Shao , S. J. Malcolmson , Org. Lett. 2020, 22, 1681–1685. 32013445 10.1021/acs.orglett.0c00342 PMC7079280

[34] H. Mitsudera , C.-J. Li , Tetrahedron Lett. 2011, 52, 1898–1900.

[35] L. Chu , F.-L. Qing , Chem. Commun. 2010, 46, 6285–6287. 10.1039/c0cc01073a 20694243

[36] W. Fu , W. Guo , G. Zou , C. Xu , J. Fluorine Chem. 2012, 140, 88–94.

[37] Referenz [11] zeigt ein isoliertes Beispiel eines ähnlichen Hydridübertragungsereignisses, allerdings auf ein β-Silicium-stabilisiertes Vinylkation.

[38a] Verwendung der Strecker-Reaktion: A. Strecker , Ann. Chem. Pharm. 1850, 75, 27–45;

[38b] J. Wang , X. Liu , X. Feng , Chem. Rev. 2011, 111, 6947–6983; 21851054 10.1021/cr200057t

[38c] K. Maruoka , T. Ooi , Chem. Rev. 2003, 103, 3013–3028; Using Radical coupling:12914490 10.1021/cr020020e

[38d] S. Ni , A. F. Garrrido-Castro , R. R. Merchant , J. N. de Gruyter , D. C. Schmitt , J. J. Mousseau , G. M. Gallego , S. Yang , M. R. Collins , J. X. Qiao , K. Yeung , D. R. Langley , M. A. Poss , P. M. Scola , T. Qin , P. S. Baran , Angew. Chem. Int. Ed. 2018, 57, 14560–14565; 10.1002/anie.201809310 PMC6352899 30212610

[38e] J. A. Bodkin , M. D. McLeod , J. Chem. Soc. Perkin Trans. 1 2002, 2733–2746; Using Biocatalysis:

[38f] S. Wu , Y. Zhou , T. Wang , H.-P. Too , D. I. C. Wang , Z. Li , Nat. Commun. 2016, 7, 11917. 27297777 10.1038/ncomms11917 PMC4911676

[39] S. Piper , N. Risch , Synlett 2004, 1489–1496.

[40] N. Risch , S. Piper , A. Winter , A. Lefarth-Risse , Eur. J. Org. Chem. 2005, 387–394.

[41] S. Dhiman , Infect. Dis. Poverty 2019, 8, 14–33. 30760324 10.1186/s40249-019-0524-x PMC6375178

[42] K. Raghavendra , T. K. Barik , B. P. Reddy , P. Sharma , A. P. Dash , Parasitol. Res. 2011, 108, 757–779. 21229263 10.1007/s00436-010-2232-0

[43] J. Gao , Z. Tian , X. Yang , Biosci. Trends. 2020, 14, 72–73. 32074550 10.5582/bst.2020.01047

